# Flavonoid Levels and Antioxidant Capacity of Mulberry Leaves: Effects of Growth Period and Drying Methods

**DOI:** 10.3389/fpls.2021.684974

**Published:** 2021-08-04

**Authors:** Lei Hu, Cheng Wang, Xiang Guo, Dekui Chen, Wei Zhou, Xiaoyang Chen, Qing Zhang

**Affiliations:** ^1^College of Forestry and Landscape Architecture, Guangdong Province Research Center of Woody Forage Engineering Technology, South China Agricultural University, Guangzhou, China; ^2^Guangdong Research and Development Center of Modern Agriculture (Woody Forage) Industrial Technology, South China Agricultural University, Guangzhou, China; ^3^Guangdong Key Laboratory for Innovative Development and Utilization of Forest Plant Germplasm, State Key Laboratory for Conservation and Utilization of Subtropical Agro-Bioresources, South China Agricultural University, Guangzhou, China

**Keywords:** mulberry leaves, flavonoids, growth period, drying methods, antioxidant activity

## Abstract

In recent years, various mulberry leaf dishes have gradually gained in popularity. The harvesting period of mulberry leaves and the preparation of dried samples are critical for the retention of flavonoid content and activity. In this study, changes in flavonoid levels in mulberry leaves during their growth period (3–6 months), and the effects of four different drying methods [sun drying (SD), air drying (AD), oven drying (OD), and freeze drying (FD)] on flavonoid accumulation and antioxidant capacity were determined. The results showed that mulberry leaves grown for 6 months had higher levels of flavonoids, and different drying methods could significantly affect the flavonoid levels and antioxidant capacity of the leaves. Air drying and FD were the best methods in terms of retaining the antioxidant activity of flavonoids, although AD had lower operating costs than FD in the production process. Therefore, to obtain a high flavonoid content and maximum antioxidant activity, AD is recommended for mulberry leaves.

## Introduction

Mulberry (*Morus alba* L.) is a woody plant of the genus *Morus* in the family Moraceae (Wang et al., 2019). Mulberry leaves are rich in nutrients and can be eaten as vegetables. Moreover, the active ingredients of mulberry leaves can be used to treat various diseases, such as hypertension, diabetes, hyperlipidemia, Alzheimer's disease, and immunomodulation (Ma et al., 2018). The popularity of mulberry may be due to it being a rich source of active substances, such as vitamins, amino acids, carotene, flavonoids, alkaloids, and polysaccharides (Rodrigues et al., 2019). In particular, flavonoids show a high therapeutic activity (Li et al., 2009).

Flavonoids are widely found in various plants, fruits, vegetables, and beverages. From a structural perspective, flavonoids can be divided into flavones, flavonols, flavanols, anthocyanins, and isoflavones (Winkel-Shirley, 2001), and are mainly present in food as glycosides and polymers (Heim et al., 2002). Flavonol glycosides, rutin, quercetin 3-(6-malonylglucoside), and isoquercitrin have been identified as the major antioxidants in ethanol extracts of *M. alba* leaves (Katsube et al., 2006). Many flavonoids exert antioxidant and free radical scavenging activity, the ability to prevent coronary heart disease, and hepatoprotective, anti-inflammatory, and anti-cancer effects (Kumar and Pandey, 2013). Flavonoids with high antioxidant activity act in a variety of ways, such as (a) can inhibit reactive oxygen species inhibition (b) leukocyte immobilization (c) inhibit nitric oxide, and (d) xanthine oxidase inhibition (Kumar and Pandey, 2013). In addition to their physiological functions in plants, flavonoids are an important part of the human diet (Procházková et al., 2011).

It is generally believed that the loss of natural antioxidants in food is related to preservation methods (Nicoli et al., 1999). Therefore, processing is required to extend shelf life. Flavonoids may be affected by various processing methods, including drying. Traditional drying methods, such as cold drying, daylight drying, hot air/oven drying, and vacuum drying, are widely used (Kamiloglu et al., 2015). In recent years, many alternative drying methods have been developed, which have the advantage of shortening the drying time, thus saving electricity and energy, and improving the quality of the final products; these methods include microwave drying, infrared drying, vacuum microwave drying, and freeze drying (FD) (Kamiloglu et al., 2015). Studies have shown that biologically active substances in natural materials are affected by high temperatures, microwave/ultraviolet radiation, and oxygen-enriched air. Therefore, during the process of preparing dry samples from natural materials, the choice of method plays a crucial role in maintaining biologically active compounds.

In recent years, targeted metabolomics methods have been widely used to analyze plant metabolomics (Yang et al., 2020). There have been a few studies on the metabolism of flavonoids in mulberry. This study was based on multiple reaction monitoring (MRM). Flavonoids were subjected to a metabolomic analysis, and metabolic changes of flavonoids in mulberry leaves were assessed at different growth stages with the application of various drying methods. The antioxidant activity of, and optimal harvesting time and drying method for, mulberry leaves were assessed.

## Materials and Methods

### Plant Materials

Mulberry leaves were harvested from an experimental field at South China Agricultural University (23.24°N, 113.64°E). The first leaf harvest was performed at 3 months (young leaves, YL), after which plants were allowed to regrow. The second leaf harvest was performed 6 months after the YL had been harvested (old leaves, OL). The samples were packed in polyamide bags and stored under vacuum.

### Preparation of Dried Samples

The harvested mulberry leaves were immediately dried to a constant weight by four different drying methods, as follows.

FD: *M. alba* samples were frozen completely in a freezer (Pilot 5-8S; Boyikang, Tianjin, China) at −60°C for 24 h to constant weight.

Oven drying (OD): *M. alba* samples were dried in a hot-air oven (DHG-9140A; Yiheng, Shanghai, China) at 65°C for 48 h to a constant weight.

Sun drying (SD): *M. alba* samples were dried under the sun for 72 h to a constant weight at 35 ± 2°C.

Air drying (AD): *M. alba* samples were dried in a shaded and ventilated place for 1 week to a constant weight at 25 ± 2°C.

### Generation of a MicroTom Metabolic Network Dataset

The samples were sent to Wuhan Metware Biotechnology Co., Ltd. (Wuhan, China) (http://www.metware.cn/) and analyzed using a flavonoid-targeting metabolomics method. In brief, each sample was crushed for 1.5 min at 30 Hz/min using a mixer mill operating at 30 Hz (MM 400; Retsch Technology, Haan, Germany) with zirconia beads. A 100 mg aliquot of the powder was extracted with 1.0 ml of 70% methanol aqueous solution at 4°C overnight. Then, the sample was centrifuged at 10,000 × *g* for 10 min, and the extract was absorbed using the CNWBOND Carbon-GCB SPE column (250 mg, 3 ml; ANPEL, Shanghai, China, http://www.anpel.com.cn/cnw) and filtrated using a microporous membrane (SCAA-104, 0.22-μm pore size; ANPEL, Shanghai, China, http://www.anpel.com.cn/) prior to ultraperformance liquid chromatography tandem mass spectrometry (UPLC-MS/MS) analysis (Chen et al., 2013). A stepwise multiple ion monitoring–enhanced product ion (MIM-EPI) strategy was used for qualitative analysis. The MS2T data were analyzed to more accurately determine precursor ion (Q1) and product ion (Q3) values, retention time (RT), and fragmentation patterns compared with those obtained by injecting standards under the same conditions, which is dependent on the availability of standards (Chen et al., 2013).

### Determination of the Moisture Content

After drying to a constant weight, the ratio of the mass of the dried sample to the mass of the original sample was determined to represent the amount of dry matter, and expressed as a percentage. After drying, samples were crushed and dried again to obtain the highest equilibrium moisture content of each drying method. A 4 g sample was accurately weighed and dried in a hot-air oven at 105°C to a constant weight (±0.02 g). The highest equilibrium moisture content was given by the ratio of moisture loss to sample weight, expressed as a percentage (Chuyen et al., 2017).

### Measurement of Antioxidant Capacity

Preparation and subsequent analysis of the extract followed the method used by He et al. (2020). The sample (0.2 g) was extracted with 10 mL methanol for 24 h in the dark, and the supernatant was then used to analyze the scavenging activities of the 2,2'-azinobis-3-ethylbenzothiazoline-6-sulfonic acid diammonium salt (ABTS) radical cation, 2,2-diphenyl-1-picrylhydrazyl (DPPH), and the ferric-reducing antioxidant power (FRAP). The absorbance was read at 517, 734, and 593 nm on microplate readers (Varioskan LUX; Thermo Fisher, Waltham, MA, United States). Trolox was used to establish a standard line, and the scavenging activity and reducing power were expressed as Trolox equivalent (mg TE/g drying sample).

### Statistical Analysis

Each sample was analyzed using three biological replicates, and the data are expressed as mean (n = 3) ± standard deviation (SD). Principal component analysis (PCA) and other multivariate statistical analysis methods were applied. The Kyoto Encyclopedia of Genes and Genomes (KEGG) pathway database (http://www.kegg.jp/kegg/pathway.html) was used to annotate the different metabolites. SPSS software (version 21.0; SPSS Inc., Chicago, IL, United States) was used to conduct Duncan's test, and *P* < 0.05 was taken to indicate a significant difference.

## Results

### Comparison of Flavonoid Metabolites in YL and OL

Dynamic changes of flavonoid metabolites in YL and OL in mulberry were detected using UPLC-MS/MS. Supplementary Figure 1 shows the total ion current (TIC) and extracted ion current (XIC); the signal was considered stable when multiple metabolites were detected in the same sample. The stability of the instrument guaranteed the repeatability and reliability of the metabolomics data.

#### Qualitative and Quantitative Analyses of Flavonid Metabolites

In MRM mode, UPLC-MS/MS was used to evaluate the compounds in the two samples.

**Figure 1:**
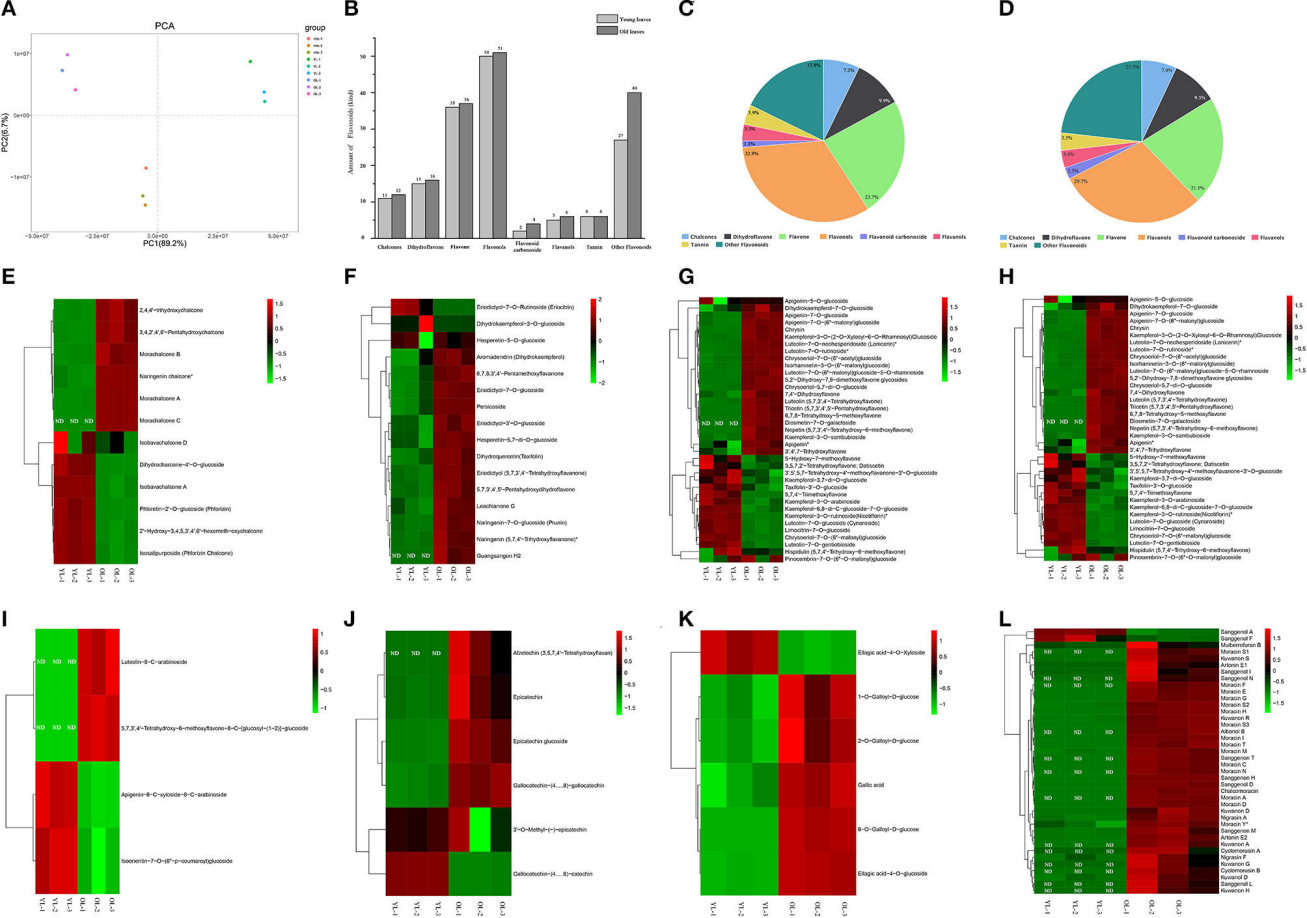
Qualitative and quantitative analyses of flavonoids metabolites in young leaves (YL) and old leaves (OL). **(A)** Principal component analysis of flavonoids metabolomics data of YL and OL. **(B)** The amount of each kind of flavonoids in YL and OL. **(C)** The ratio of each type of flavonoids in YL. **(D)** The ratio of each type of flavonoids in OL. **(E–L)** Heatmap of flavonoids metabolomics data of YL and OL. **(E)** Chalcones, **(F)** Dihydroflavone, **(G)** Flavone, **(H)** Flavonols, **(I)** Flavonoid carbonoside, **(J)** Flavanols, **(K)** Tannin, and **(L)** Other flavonoids. The heat map showed the relative content of metabolites, where each line represents a metabolite. The red and green segments represent a relatively high and low abundance of metabolites, respectively. ND, Not Found.

PCA was conducted to confirm the metabolic differences between the YL and OL samples, and the variance explained by the first two principal components was determined (89.2 and 6.7%, respectively). The results showed that there were different metabolites in the YL and OL sample groups, and each sample formed a separate cluster (Figure 1A). The significant differences between the samples could inform subsequent qualitative and quantitative analyses. The qualitative analysis revealed that a total of 172 flavonoids metabolites (12 chalcones, 16 dihydroflavones, 37 flavones, 51 flavonols, four flavonoid carbonosides, six flavanols, six tannins, and 40 other flavonoids) were identified/annotated in OL, while 152 flavonoid metabolites (11 chalcones, 15 dihydroflavones, 36 flavones, 50 flavonols, two flavonoid carbonosides, five flavanols, six tannins, and 27 other flavonoids) were identified/annotated in YL (Figures 1B–D). All of the flavonoids in YL were also present in OL. In addition, a hierarchical cluster analysis was performed to assess the accumulation patterns of the various flavonoid metabolites in OL and YL (Figures 1E–L).

#### Identification, Functional Annotation, and KEGG Enrichment Analysis of Differentially Accumulated Metabolites

**Figure 2:**
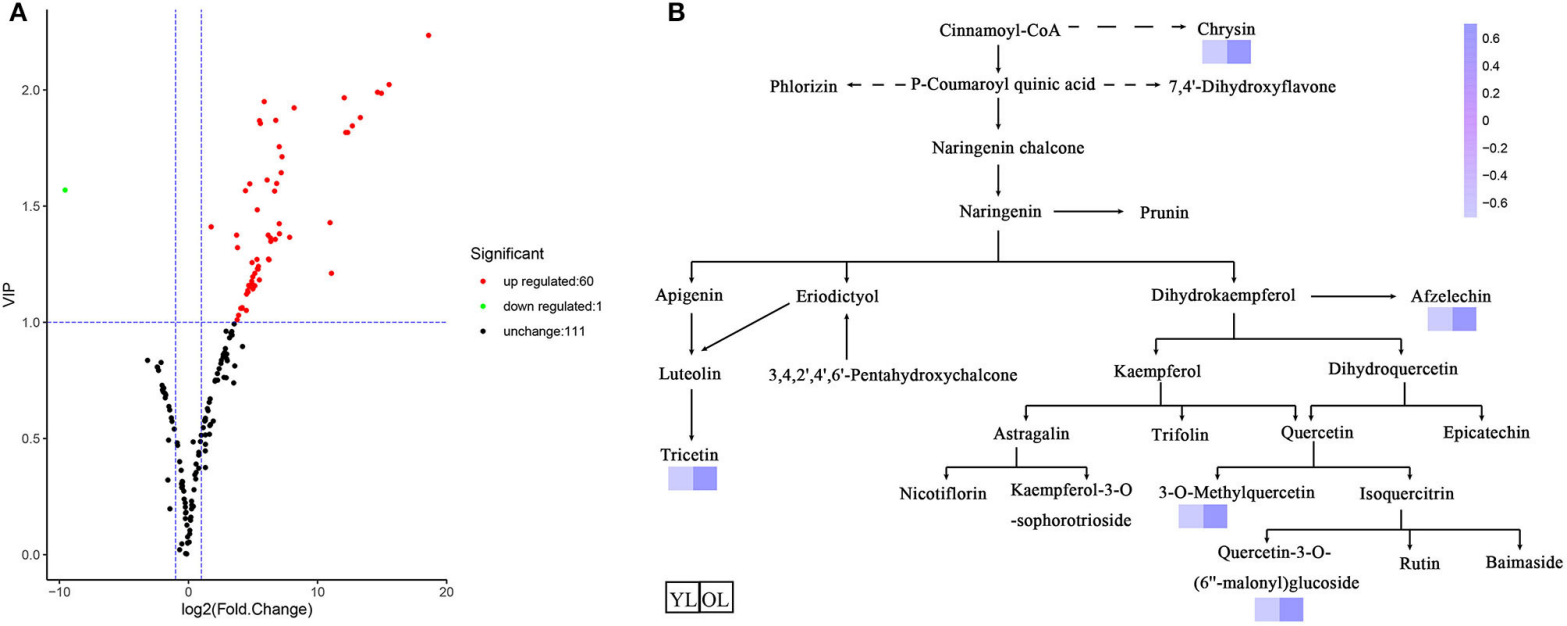
Differentially accumulated metabolites between YL and OL. **(A)** The volcano plot shows the flavonoids metabolites accumulate levels between YL and OL. **(B)** Schematic representation of flavonoid biosynthesis. The dashed arrows represent multiple enzymatic steps. A darker color represents an increase in metabolites.

Differentially accumulated metabolites (DAMs) were defined as those showing a variable importance in projection (VIP) value ≥ 1 and a fold-change of ≥ 2 or ≤ 0.5 between OL and YL (*P* < 0.05). A total of 61 DAMs were identified. Volcano plots were generated to represent the DAMs between OL and YL (Figure 2A): 60 (three chalcones, three dihydroflavones, eight flavones, nine flavonols, two flavonoid carbonosides, two flavanols, one tannin, and 32 other flavonoids) of 61 (two chalcones, three dihydroflavones, 10 flavones, nine flavonols, two flavonoid carbonosides, two flavanols, and 32 other flavonoids) compounds were up-regulated (98.36%) and one (flavone) of 60 was down-regulated (1.64%).

The KEGG database was used to annotate the functions of the DAMs between OL and YL; the metabolic pathway analysis results are shown in Figure 2B. Only five (chrysin, afzelechin, 3-O-methylquercetin, quercetin-3-O-(6”-malonyl)glucoside, and tricetin) of the 61 flavonoids were annotated by KEGG. The KEGG classification and enrichment results indicated that the differences in flavonoid metabolites between YL and OL were related to flavonoid biosynthesis, isoflavonoid biosynthesis, and flavone and flavonol biosynthesis pathways. All five of these flavonoids displayed an upward expression trend in the OL group and were located in the downstream region of each metabolic pathway.

### Effects of Different Drying Methods on the Flavonoids in Mulberry Leaves

Mulberry leaves are known to contain high levels of flavonoids. How best to ensure the retention of flavonoids is a hot topic in food processing research. To preserve the flavonoids in mulberry leaves, we examined the effects of four different drying methods on the accumulation of flavonoids in YL and OL.

#### Moisture Content of Dried Leaves From Different Drying Methods

The moisture content of dried samples was affected by the drying temperature, and was related to the growth period of the leaves. Compared to the instrumental drying methods (OD and FD), the natural drying methods (AD and SD) were associated with a higher percentage leaf water loss, that is, the dry matter content of the leaf was low (Supplementary Table 1).

The highest equilibrium moisture content of the sample after drying ranged from 2.23 to 6.03% (Supplementary Table 1). The moisture content in the leaves obtained by all drying methods was in the safe range for dried fruit and plant material storage, and allowed subsequent analysis of flavonoids and antioxidant properties.

#### Metabolite Profiling Analysis

In MRM mode, UPLC-MS/MS was used to evaluate the compounds in the four YL and OL samples.

**Figure 3:**
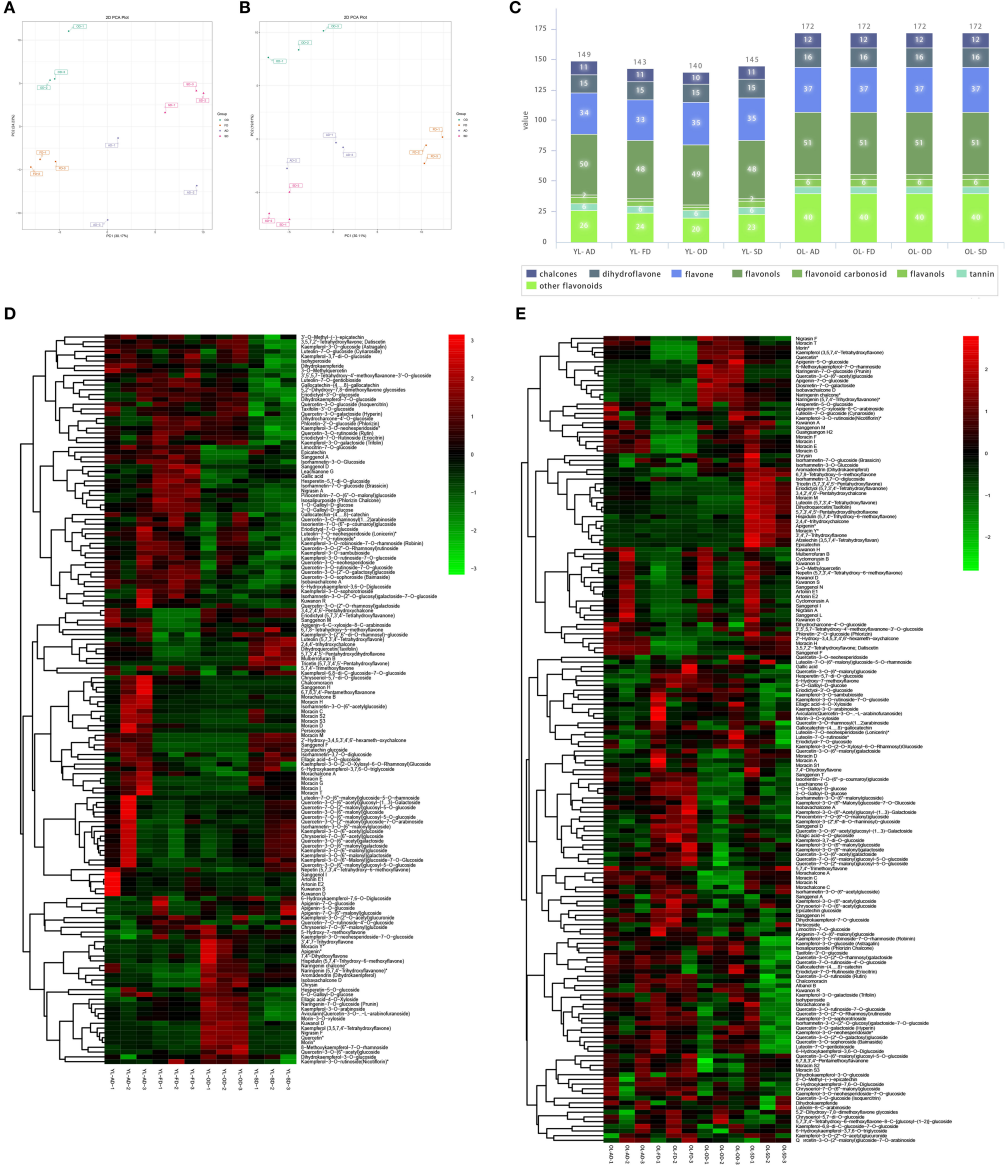
The effect of drying methods on flavonoid metabolites of YL and OL. **(A)** Principal component analysis (PCA) on the effect of drying methods on flavonoids metabolites of YL. **(B)** PCA on the effect of drying methods on flavonoids metabolites of OL. **(C)** Stacked graphs of the amounts of various flavonoids in different drying methods of YL and OL. **(D)** Heatmap of flavonoids in different drying methods of YL. **(E)** Heatmap of flavonoids in different drying methods of OL.

In YL, PCA showed that the metabolites associated with the four drying methods were separated from each other into individual clusters. The variance explained by the first two main components was 30.17 and 24.23%, respectively (Figure 3A). After quality verification, 149, 143, 140, and 145 flavonoid metabolites were identified/labeled in the AD, FD, OD, and SD groups, respectively (Figure 3C). A hierarchical cluster analysis was also performed (Figure 3D).

In OL, PCA showed that the metabolites associated with the four drying methods were separated from each other into individual clusters, and the variance explained by the first two main components was 47.2 and 31.0%, respectively (Figure 3B). A total of 172 flavonoid metabolites were identified/annotated in each group (Figure 3C). A hierarchical cluster analysis was also performed (Figure 3E).

#### Identification of DAMs

**Figure 4:**
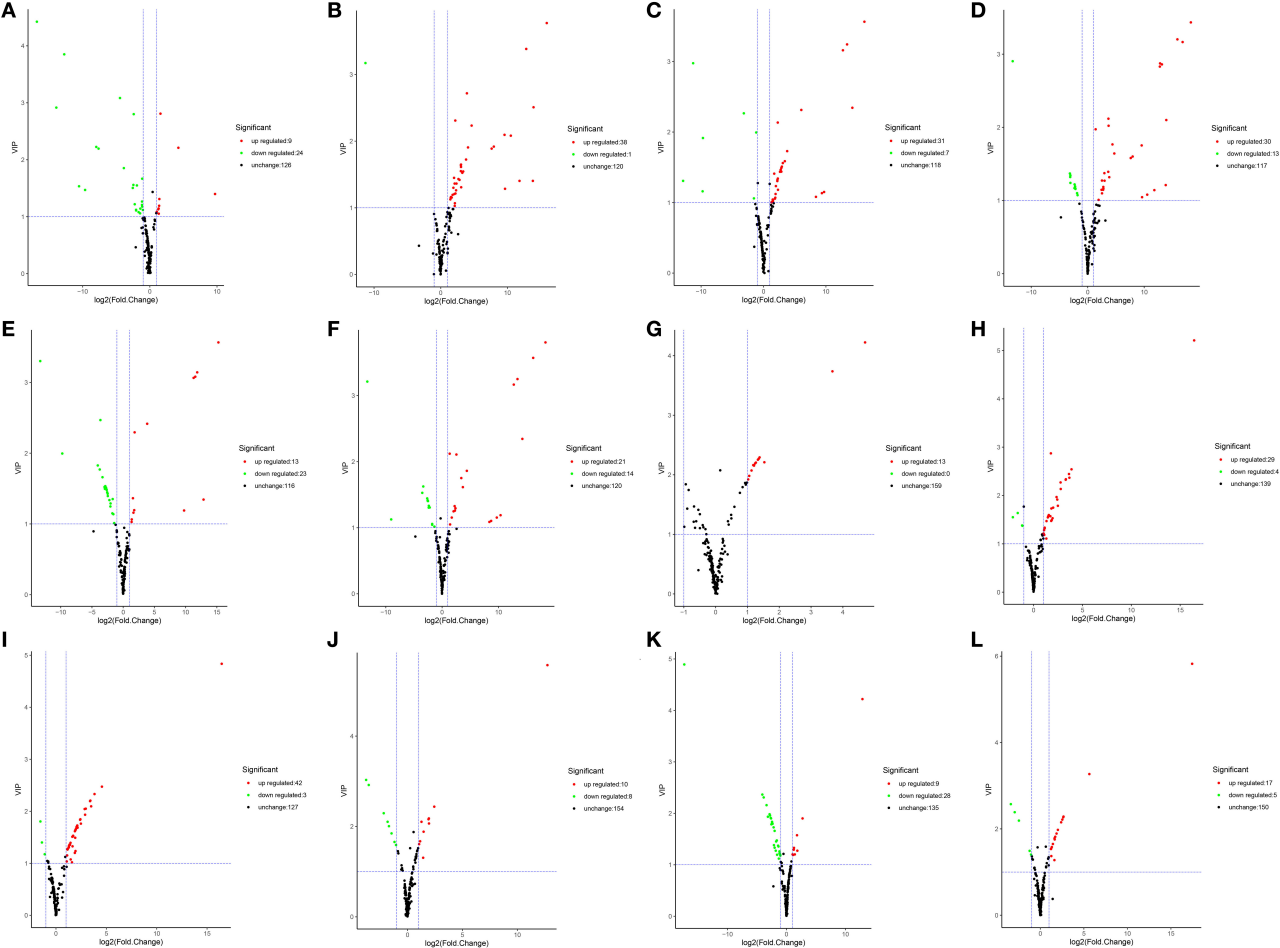
The volcano plot shows the accumulate levels of flavonoid metabolites in different drying methods in YL or OL. **(A–F)** in YL. **(A)** Air dried (AD) vs. sun dried (SD), **(B)** freeze dried (FD) vs. AD, **(C)** FD vs. SD, **(D)** oven dried (OD) vs. AD, **(E)** OD vs. FD, **(F)** OD vs. SD. **(G–L)** in YL. **(G)** AD vs. SD, **(H)** FD vs. AD, **(I)** FD vs. SD, **(J)** OD vs. AD, **(K)** OD vs. FD, and **(L)** OD vs. SD.

Differentially accumulated metabolites were defined as those showing a VIP value ≥ 1 and a fold-change of ≥ 2 or ≤ 0.5 between YL and OL (OD vs. FD, AD vs. FD, SD vs. FD, OD vs. SD, OD vs. AD, and AD vs. SD) (*P* < 0.05). A total of 61 DAMs were identified. Volcano plots were generated to represent the DAMs and show the significant differences. A hierarchical cluster analysis was performed to assess the DAM accumulation patterns (Figure 4).

In YL (Figures 4A–F), for AD vs. SD, nine of 33 flavonoids were up-regulated (27.3%) and 24 of 33 were down-regulated (72.7%); for AD vs. FD, 38 of 39 were up-regulated (97.4%) and 1 of 39 was down-regulated (2.6%); for SD vs. FD, 31 of 38 were up-regulated (81.6%) and 7 of 38 were down-regulated (18.4%); for OD vs. AD, 30 of 43 were up-regulated (69.8%) and 13 of 43 were down-regulated (30.2%); for OD vs. FD, 13 of 36 were up-regulated (36.1%) and 23 of 36 were down-regulated (63.9%); and for OD vs. SD, 21 of 35 were up-regulated (60.0%) and 14 of 35 were down-regulated (40.0%). A total of 64 DAMs were identified. Compared to the other three groups, the SD group had 13 types of significantly up-regulated flavonoids, while the OD group had 17, the AD group had 28, and the FD group had 6. The experimental results showed that the accumulation of flavonoids followed the order of AD > OD > SD > FD in YL.

In OL (Figures 4G–L), for AD vs. SD, 13 of 13 flavonoids were up-regulated (100.0%) and none were down-regulated (0.0%); for AD vs. FD, 29 of 33 were up-regulated (87.9%) and four of 33 were down-regulated (12.1%); for SD vs. FD, 42 of 45 were up-regulated (93.3%) and three of 45 were down-regulated (6.7%); for OD vs. AD, 10 of 18 were up-regulated (55.6%) and eight of 18 were down-regulated (44.4%); for OD vs. FD, nine of 37 were up-regulated (24.3%) and 28 of 37 were down-regulated (75.7%); and for OD vs. SD, 17 of 22 were up-regulated (77.3%) and 5 of 22 were down-regulated (22.7%). A total of 61 DAMs were identified. Compared to the other three groups, the SD group had 23 types of significantly up-regulated flavonoids, while the OD group had 18, the AD group had 13, and the FD group had 8. The experimental results showed that the accumulation of flavonoids followed the order of SD > OD > AD > FD in OL.

#### Functional Annotation and KEGG Enrichment Analysis of DAMs

**Figure 5:**
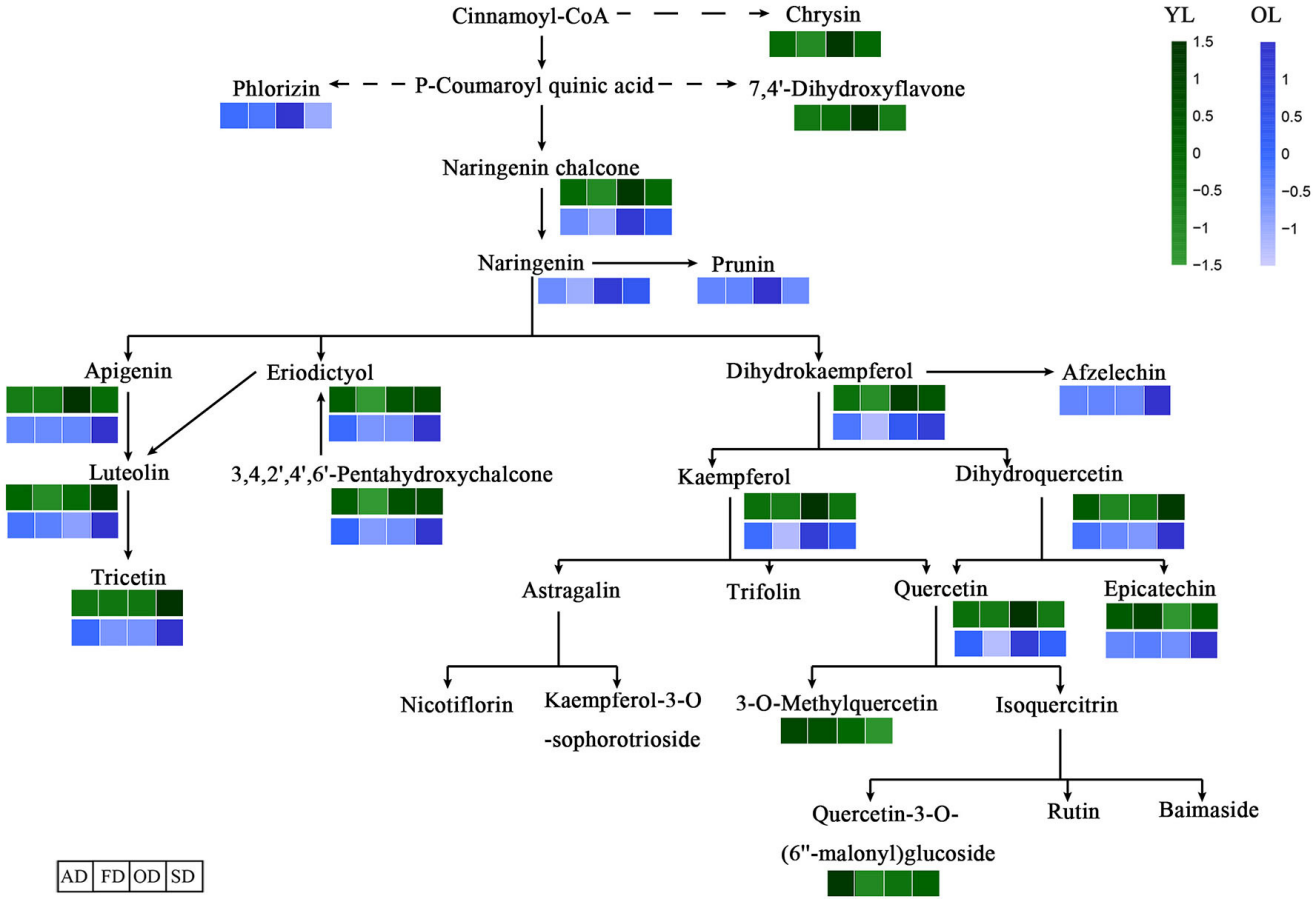
Effects of different drying methods on the accumulate of flavonoids. The dashed arrows represent multiple enzymatic steps. A darker color represents an increase in metabolites.

The DAMs associated with the four drying methods were functionally annotated using the KEGG database. The annotation results regarding the differentially expressed metabolites in the metabolic pathways between the four drying methods are shown in Figure 5. In total, 15 of the 64 flavonoids in YL and 15 of the 61 in OL were annotated by KEGG. The results of the KEGG enrichment analysis showed that DAMs were annotated in the flavonoid biosynthesis, isoflavonoid biosynthesis, and flavone and flavonol biosynthesis pathways. All 15 flavonoids in OL were located in the midstream of each metabolic pathway.

### Effect of Drying Methods on Antioxidant Capacity

The antioxidant activity of flavonoids, which is mainly due to free radical scavenging, is important for health care applications (Yu et al., 2020). In this study, three measurement methods were used to evaluate the effect of the different drying methods on the overall antioxidant capacity of mulberry leaves. The FRAP assays were conducted to measure the trivalent iron reduction ability of the antioxidants. The DPPH radical scavenging ability (DRSC) and ABTS radical scavenging ability (ARSC) analyses were based on the ability of antioxidants to transfer single electrons to ABTS/DPPH radicals forming electron pairs.

#### Ferric-Reducing Antioxidant Power

The results of the FRAP assays of the mulberry extracts obtained using four drying methods are shown in Table 1. The levels of phytochemical compounds were highest in YL-AD and YL-FD, with a FRAP of 36.39 and 36.54 mg TE/g dry sample, respectively, followed by YL-SD and YL-OD (32.83 and 31.58 mg TE/g dry sample, respectively); OL-SD, OL-AD, and OL-FD had a lower FRAP (14.58, 17.33, and 23.43 mg TE/g dry sample, respectively), while the lowest value was observed for OL-OD (12.98 mg TE/g dry sample).

**Table 1:** Antioxidant capacity of mulberry leaves dried by four different methods in YL and OL.

**Drying method**	**ARSC (mg TE/g)**	**DRSC (mg TE/g)**	**FRAP (mg TE/g)**
YL-OD	57.03 ± 1.33^b^^*^	19.59 ± 0.71^b^	31.58 ± 1.18^b^
YL-FD	62.58 ± 1.53^a^	21.40 ± 0.55^a^	36.54 ± 1.16^a^
YL-AD	63.92 ± 0.95^a^	21.76 ± 0.60^a^	36.39 ± 0.50^a^
YL-SD	59.24 ± 1.09^b^	20.89 ± 0.44^ab^	32.83 ± 0.40^b^
OL-OD	38.20 ± 2.44^d^	9.33 ± 0.40^e^	12.98 ± 0.23^e^
OL-FD	62.24 ± 2.32^a^	17.19 ± 0.58^c^	23.43 ± 0.44^c^
OL-AD	47.52 ± 1.98^c^	12.85 ± 0.87^d^	17.33 ± 1.40^d^
OL-SD	45.85 ± 0.64^c^	11.78 ± 0.54^d^	14.58 ± 0.66^e^

*n = 3. TE, Trolox equivalents*.

* *Different letters within the same column denote significant differences between groups (P < 0.05)*.

#### ABTS Radical Scavenging Ability

The ARSCs of mulberry leaves after applying the four drying methods are shown in Table 1. The YL-AD, YL-FD, and OL-FD had the highest ARSCs (63.92, 62.58, and 62.24 mg TE/g dried sample, respectively), followed by YL-SD and YL-OD (59.24 and 57.03 mg TE/g dried sample, respectively), and OL-SD and OL-AD (45.85 and 47.52 mg TE/g dried sample, respectively); the lowest value was observed for OL-OD (38.20 mg TE/g dried sample).

#### DPPH Radical Scavenging Ability

The DRSCs of the mulberry extracts after applying the four drying methods are shown in Table 1. As with the levels of phytochemical compounds, YL-AD and YL-FD had the highest DRSCs (21.76 and 21.40 mg TE/g dried sample, respectively), followed by YL-SD, YL-OD, and OL-FD (20.89, 19.95, and 17.19 mg TE/g dried sample, respectively). The lowest value was observed for OL-OD (9.33 mg TE/g dried sample), followed by OL-SD and OL-AD (11.78 and 12.85 mg TE/g dried sample, respectively).

The above results show that, after applying the same drying method, the antioxidant capacity of YL was higher than that of the OL (Table 1). Among the different drying methods used in this study, AD at 25°C and FD at −60°C resulted in the highest flavonoid content (YL-AD ≈ YL-FD > YL-SD ≈ YL-OD > OL-FD > OL-AD ≈ OL-SD > OL-OD) (Table 1).

## Discussion

Many edible mulberry leaf products have been developed in various regions, including mulberry leaf tea, mulberry leaf powder, and mulberry leaf juice (Yu et al., 2018). Through ongoing research, mulberry leaf edible products are now marketed as a functional health food. The levels of effective functional components in mulberry leaves vary by mulberry variety, harvesting time, and processing technology. Using an appropriate processing technology improves the preservation of functional components in mulberry leaves, and efficient absorption thereof by the human body.

Previous studies have focused on the isolation, identification, and accumulation of flavonoid components in various mulberry species (Ju et al., 2018). Very few studies have considered mulberry metabolomics. Our MicroTom Metabolic Network (MMN) dataset supplements existing mulberry flavonoid metabolomics data. Our results show that flavonoid accumulation differs throughout the mulberry growth period.

Through comprehensive analysis of the MMN dataset, we constructed a map of the changes in flavonoid metabolites during the growth of mulberry leaves (Figure 2B). In total, 172 metabolites were identified, including 22 novel flavonoids (Figure 1), and 38 types of flavonoids were significantly up-regulated (Figure 2A). Among the 61 kinds of flavonoids, five were shown by KEGG analysis to function downstream of the flavonoid biosynthesis, isoflavonoid biosynthesis, and flavone and flavonol biosynthesis pathways.

The reasons for the increase in accumulation and types of flavonoids in OL might be as follows. First, there were ripening-dependent changes in total flavonoid content (Bhandari et al., 2013). Through PCA, as well as DAM and KEGG analyses, significant variations in the accumulates of flavonoids during the growing months were identified, where two flavones (chrysin and tricetin), two flavonols (3-O-methylquercetin and quercetin-3-O-(6”-malonyl)glucoside), and one flavanol (afzelechin) were used as growth markers. The experimental results of Sun et al. (2019) were similar to ours. The flavonoid *Zanthoxylum bungeanum* (Fugu) likely accumulates during the growth period. Ganzon et al. (2018) found that flavonoids accumulate in apical leaves at significantly higher levels than in lower leaves. Therefore, the difference in flavonoid content that we observed between OL and YL might be related to the harvesting location.

Secondary metabolites can exert a protective action by reducing plant damage caused by biological stresses (Liu et al., 2015). The biosynthesis of flavonoids is regulated by multiple factors, among which temperature is the primary factor (Gouot et al., 2019). When subjected to heat stress, plants protect themselves by producing natural antioxidants, such as flavonoids and phenols (Ghasemzadeh et al., 2010).

In addition to regulating the growth and development of plants, phytohormones also participate in plant secondary metabolism (Zhao et al., 2005). The synthesis of flavonoids is affected by plant growth regulators, such as auxins, cytokinins, gibberellins, and abscisic acid (Guo et al., 2020).

Preservation methods, including drying, can deplete natural antioxidants in foods (Nicoli et al., 1999). Food processing also has an impact on food composition, which must be considered for accurate evaluation of human health research data (Nicoli et al., 1999). Drying is one of the methods.

Differences in moisture content among the four different drying methods could be due to differences in temperature, air relative humidity, and airflow/speed (Nguyen et al., 2015). The results of a metabolomic analysis showed that the accumulation of flavonoids in OL were higher than that in YL. However, *in vitro* experiments showed that YL had better antioxidant properties than OL. Moreover, after experiencing stress, the levels of flavonoids in plants first increase and then decrease (Dalian and Yuxin, 2013). In this study, the levels of flavonoids in OL displayed a downward trend during the experiment, and were relatively low overall. During plant growth, nutrients and energy in the OL may be transferred to developing leaves, enabling growth, reproduction, and defense despite limited nutrient availability (Chen et al., 2019).

Studies have shown that the levels of flavonoids follow an upward trend during the drying of mulberry leaves (Zhang, 2015). Sung et al. (2019) found that flavonoid levels, especially of the potent aglycones, increase in peel after heat treatment. Our experimental results showed that the levels of flavonoids followed the order of AD > OD > SD > FD in YL, SD > OD > AD > FD in OL. Chuyen et al. (2017) obtained similar results; a high carotenoid content was obtained by drying at 70°C, while the carotenoid content was lower in Gac peel after low-temperature drying and FD. After the leaves had been removed, mesophyll cells survived and were under stress for a certain period of time. The AD process at room temperature resulted in gradual loss of moisture. Mesophyll cells survived and could still carry out complex metabolic activities, which might include converting certain intermediate products into flavonoids and thus increasing the accumulate of flavonoids. However, the temperature of the FD process was low, so the metabolism of mesophyll cells was inhibited, and substance changes were not obvious. Therefore, the levels of flavonoids in mulberry leaves did not change significantly (Zhao et al., 2020). However, due to the influence of high temperature, the activity of flavonoids could be inhibited. And drying at low temperature, such as room temperature, could better preserve the biological activity of flavonoids. Freeze drying can effectively preserve antioxidants. Pérez-Gregorio et al. (2011) found that the antioxidant activity of onions after FD was not significantly different from that of fresh samples. However, Chiang et al. (2008) found that the antioxidant activity of water chestnut after FD was higher than that of fresh samples. As shown in Table 1, the antioxidant activity of mulberry leaves after the application of different drying methods was affected by temperature: the higher the temperature, the lower the antioxidant activity. The decrease in antioxidant properties in mulberry leaves was related to thermal degradation and the content of polyphenol oxidase. The common drying methods will adversely affect the stability of antioxidant substances in mulberry leaves through thermal degradation or the release of polyphenol oxidase. The decrease in antioxidant activity of mulberry leaves after drying at 65°C can be attributed to the thermal degradation of phenolic compounds. The peroxidase may still retain some activity during the drying process (Tan et al., 2015). Ma et al. (2018) showed that as the temperature increased, the yield of polysaccharides from mulberry leaves decreased. Polysaccharides have a synergistic effect with glycolysis on the biosynthesis of flavonoids (Luo et al., 2020).

Flavonoids, as secondary plant metabolites, scavenge or prevent the formation of ROS by chelating transition metal ions (such as iron and copper), and therefore show high levels of antioxidant activity (Zhang et al., 2016). Almost every group of flavonoids has antioxidant activity (Panche et al., 2016), with complementary and overlapping mechanisms of action (Ren et al., 2003).

The antioxidant activity of flavonoids, and their metabolites, *in vitro* depends on the arrangement of functional groups around the nuclear structure (Heim et al., 2002). Quercetin glycosides play a major role in the antioxidant activity of mulberry leaves (Katsube et al., 2009). Chrysin, luteolin, and apigenin may show antioxidant activity at low concentrations (Singh et al., 2014). Epicatechin and rutin are strong free radical scavengers and lipid peroxidation inhibitors *in vitro* (Kerry and Abbey, 1997). Overall, as shown in Figure 5 and Table 1, the highest levels of quercetin-3-O-(6”-malonyl)glucoside, 3-O-methylquercetin, epicatechin, luteolin, and chrysin were found in YL-AD, while epicatechin and 3-O-methylquercetin levels were highest in the YL-SD group (indicating a higher antioxidant activity than the other groups). In addition, although the levels of luteolin and quercetin in the OD group were significantly up-regulated compared with the other groups, the higher drying temperature might have a greater impact on their biological activity (Kamiloglu et al., 2015). Antioxidant activity was not only related to the relative levels of individual flavonoids, but also to the types thereof. Other studies showed that the effects of drying methods on flavonoid levels also vary among plant varieties. AD and FD not only better preserved the antioxidant activity of flavonoids, but also increased the accumulation thereof. In addition to flavonoids, carotenoids (Nguyen et al., 2015), ascorbic acid, phenolic acids (Orsavová et al., 2019), and polysaccharides (Ma et al., 2018) also display strong antioxidant activity. The higher antioxidant activity of YL-AD and YL-FD may be due to better preservation of other substances with antioxidant activity, as well of flavonoid activity.

## Conclusions

The levels and antioxidant activity of flavonoids in mulberry leaves were affected by growth time and drying method. The optimal accumulation was observed in mulberry leaves that had a relatively long growth period or were in the early stage of environmental stress exposure. Among the four drying methods investigated, the level of flavonoids was higher in the AD and SD groups, but the AD and FD groups could better retain the antioxidant activity of flavonoids. With consideration of costs, it is recommended that AD be utilized at room temperature to maximize the antioxidant capacity of mulberry leaves.

## Data Availability Statement

The original contributions presented in the study are included in the article/Supplementary Material, further inquiries can be directed to the corresponding author/s.

## Author Contributions

LH, CW, and XG: conceived and designed the experiments. LH and DC: performed the experiment and analyzed the data. LH and QZ: prepared the manuscript. XC, WZ, and QZ: reviewed the final manuscript. All authors contributed to the article and approved the submitted version.

## Conflict of Interest

The authors declare that the research was conducted in the absence of any commercial or financial relationships that could be construed as a potential conflict of interest.

## Publisher's Note

All claims expressed in this article are solely those of the authors and do not necessarily represent those of their affiliated organizations, or those of the publisher, the editors and the reviewers. Any product that may be evaluated in this article, or claim that may be made by its manufacturer, is not guaranteed or endorsed by the publisher.

## References

[B1] Bhandari S. R. Jung B. Baek H. Lee Y. (2013). Ripening-dependent changes in phytonutrients and antioxidant activity of red pepper (*Capsicum annuum* L.) fruits cultivated under open-field conditions. Hortscience 48, 1275–1282. 10.21273/HORTSCI.48.10.1275

[B2] Chen Q. Shinozaki D. Luo J. Pottier M. Have M. Marmagne A.. (2019). Autophagy and nutrients management in plants. Cells 8:1426. 10.3390/cells8111426 PMC6912637 31726766

[B3] Chen W. Gong L. Guo Z. Wang W. Zhang H. Liu X.. (2013). A Novel integrated method for large-scale detection, identification, and quantification of widely targeted metabolites: application in the study of rice metabolomics. Mol. Plant 6, 1769–1780. 10.1093/mp/sst080 23702596

[B4] Chiang P. Y. Ciou J. Y. Hsieh L. C. (2008). Antioxidant activity of phenolic compounds extracted from fresh and dried water caltrop pulp (*Trapa taiwanensis* Nakai). J. Food Drug Analy. 16, 66–73. 10.38212/2224-6614.2351

[B5] Chuyen H. V. Roach P. D. Golding J. B. Parks S. E. Nguyen M. H. (2017). Effects of four different drying methods on the carotenoid composition and antioxidant capacity of dried Gac peel. J. Sci. Food Agricult. 97, 1656–1662. 10.1002/jsfa.7918 27435184

[B6] Dalian C. Yuxin M. (2013). Seasonal changes and response to stress of total flavonoids content of *Farfujium japonicum* (in Chinese). J. Zhejiang Univer. 42, 319–325. 10.3785/j.issn.1008-9292.2013.03.011 23801621

[B7] Ganzon J. G. Chen L. Wang C. (2018). 4-O-Caffeoylquinic acid as an antioxidant marker for mulberry leaves rich in phenolic compounds. J. Food Drug Analy. 26, 985–993. 10.1016/j.jfda.2017.11.011 29976416 PMC9303035

[B8] Ghasemzadeh A. Jaafar H. Z. E. Rahmat A. Wahab P. E. M. Halim M. R. A. (2010). Effect of different light intensities on total phenolics and flavonoids synthesis and anti-oxidant activities in young ginger varieties (*Zingiber officinale* Roscoe). Int. J. Mol. Sci. 11, 3885–3897. 10.3390/ijms11103885 21152306 PMC2996797

[B9] Gouot J. C. Smith J. P. Holzapfel B. P. Walker A. R. Barril C. (2019). Grape berry flavonoids: a review of their biochemical responses to high and extreme high temperatures. J. Exp. Botany. 70, 397–423. 10.1093/jxb/ery392 30388247

[B10] Guo J. Zhou X. Wang T. Wang G. Cao F. (2020). Regulation of flavonoid metabolism in ginkgo leaves in response to different day-night temperature combination. Plant Physiol. Biochem. 147, 133–140. 10.1016/j.plaphy.2019.12.009 31862579

[B11] He L. Lv H. Chen N. Wang C. Zhou W. Chen X.. (2020). Improving fermentation, protein preservation and antioxidant activity of *Moringa oleifera* leaves silage with gallic acid and tannin acid. Bioresour. Techn. 297:122390. 10.1016/j.biortech.2019.122390 31740244

[B12] Heim K. E. Tagliaferro A. R. Bobilya D. J. (2002). Flavonoid antioxidants: chemistry, metabolism and structure-activity relationships. J. Nutrit. Biochem. 13, 572–584. 10.1016/S0955-2863(02)00208-5 12550068

[B13] Ju W. Kwon O. Kim H. Sung G. Kim H. Kim Y. (2018). Qualitative and quantitative analysis of flavonoids from 12 species of Korean mulberry leaves. J. Food Sci. Techn. 55, 1789–1796. 10.1007/s13197-018-3093-2 29666531 PMC5897299

[B14] Kamiloglu S. Toydemir G. Boyacioglu D. Beekwilder J. Hall R. D. Capanoglu E. (2015). A review on the effect of drying on antioxidant potential of fruits and vegetables. Crit. Rev. Food Sci. Nutrit. 56, S110–S129. 10.1080/10408398.2015.1045969 26191781

[B15] Katsube T. Imawaka N. Kawano Y. Yamazaki Y. Shiwaku K. Yamane Y. (2006). Antioxidant flavonol glycosides in mulberry (*Morus alba* L.) leaves isolated based on LDL antioxidant activity. Food Chem. 97, 25–31. 10.1016/j.foodchem.2005.03.019

[B16] Katsube T. Tsurunaga Y. Sugiyama M. Furuno T. Yamasaki Y. (2009). Effect of air-drying temperature on antioxidant capacity and stability of polyphenolic compounds in mulberry (*Morus alba* L.) leaves. Food Chem. 113, 964–969. 10.1016/j.foodchem.2008.08.041

[B17] Kerry N. L. Abbey M. (1997). Red wine and fractionated phenolic compounds prepared from red wine inhibit low density lipoprotein oxidation *in vitro*. Atherosclerosis 135, 93–102. 10.1016/s0021-9150(97)00156-1 9395277

[B18] Kumar S. Pandey A. K. (2013). Chemistry and biological activities of flavonoids: an overview. Scient. World 2013, 162716–162750. 10.1155/2013/162750 24470791 PMC3891543

[B19] Li W. Li T. Tang K. (2009). Flavonoids from mulberry leaves by microwave-assisted extract and anti-fatigue activity. Afric. J. Agricult. Res. 4, 898–902. 10.5897/AJAR

[B20] Liu B. Lei C. Shu T. Zhang Y. Jin J. Li S.. (2015). Effects of low-temperature stress on secondary metabolism in mosses exposed to simulated N deposition. Plant Ecol. Div. 8, 415–426. 10.1080/17550874.2015.1010187

[B21] Luo Y. Ge C. Ling Y. Mo F. Yang M. Jiang L.. (2020). ABA and sucrose co-regulate strawberry fruit ripening and show inhibition of glycolysis. Mol. Genet. Genom. 295, 421–438. 10.1007/s00438-019-01629-w 31807909

[B22] Ma Q. Santhanam R. K. Xue Z. Guo Q. Gao X. Chen H. (2018). Effect of different drying methods on the physicochemical properties and antioxidant activities of mulberry leaves polysaccharides. Int. J. Biol. Macromol. 119, 1137–1143. 10.1016/j.ijbiomac.2018.08.023 30098363

[B23] Nguyen V. T. Van Vuong Q. Bowyer M. C. Van Altena I. A. Scarlett C. J. (2015). Effects of different drying methods on bioactive compound yield and antioxidant capacity of *Phyllanthus amarus*. Drying Techn. 33, 1006–1017. 10.1080/07373937.2015.1013197

[B24] Nicoli M. C. Anese M. Parpinel M. (1999). Influence of processing on the antioxidant properties of fruit and vegetables. Trends Food Sci. Techn. 10, 94–100. 10.1016/S0924-2244(99)00023-0

[B25] Orsavová J. Hlaváčová I. Mlček J. Snopek L. Mišurcov,á L. (2019). Contribution of phenolic compounds, ascorbic acid and vitamin E to antioxidant activity of currant (*Ribes* L.) and gooseberry (*Ribes uva-crispa* L.) fruits. Food Chem. 284, 323–333. 10.1016/j.foodchem.2019.01.072 30744864

[B26] Panche A. N. Diwan A. D. Chandra S. R. (2016). Flavonoids: an overview. J. Nutr. Sci. 5:e47. 10.1017/jns.2016.41 28620474 PMC5465813

[B27] Pérez-Gregorio M. R. Regueiro J. González-Barreiro C. Rial-Otero R. Simal-Gándara J. (2011). Changes in antioxidant flavonoids during freeze-drying of red onions and subsequent storage. Food Control. 22, 1108–1113. 10.1016/j.foodcont.2011.01.006

[B28] Procházková D. Boušová I. Wilhelmová N. (2011). Antioxidant and prooxidant properties of flavonoids. Fitoterapia 82, 513–523. 10.1016/j.fitote.2011.01.018 21277359

[B29] Ren W. Qiao Z. Wang H. Zhu L. Zhang L. (2003). Flavonoids: promising anticancer agents. Med. Res. Rev. 23, 519–534. 10.1002/med.10033 12710022

[B30] Rodrigues E. Marcelino G. Silva G. Figueiredo P. Garcez W. Corsino J.. (2019). Nutraceutical and medicinal potential of the *Morus* species in metabolic dysfunctions. Int. J. Mol. Sci. 20:301. 10.3390/ijms20020301 30646503 PMC6358891

[B31] Singh M. Kaur M. Silakari O. (2014). Flavones: an important scaffold for medicinal chemistry. Europ.J. Med. Chem. 84, 206–239. 10.1016/j.ejmech.2014.07.013 25019478

[B32] Sun L. Yu D. Wu Z. Wang C. Yu L. Wei A.. (2019). Comparative transcriptome analysis and expression of genes reveal the biosynthesis and accumulation patterns of key flavonoids in different varieties of *Zanthoxylum bungeanum* leaves. J. Agricult. Food Chem. 67, 13258–13268. 10.1021/acs.jafc.9b05732 31714769

[B33] Sung J. Suh J. H. Wang Y. (2019). Effects of heat treatment of mandarin peel on flavonoid profiles and lipid accumulation in 3T3-L1 adipocytes. J. Food Drug Analy. 27, 729–735. 10.1016/j.jfda.2019.05.002 31324288 PMC9307040

[B34] Tan J. J. Y. Lim Y. Y. Siow L. F. Tan J. B. L. (2015). Effects of drying on polyphenol oxidase and antioxidant activity of *Morus alba* leaves. J. Food Process. Preserv. 39, 2811–2819. 10.1111/jfpp.12532

[B35] Wang Y. Chen X. Wang C. He L. Zhou W. Yang F.. (2019). The bacterial community and fermentation quality of mulberry (*Morus alba*) leaf silage with or without *Lactobacillus casei* and sucrose. Bioresour. Techn. 293:122059. 10.1016/j.biortech.2019.122059 31476563

[B36] Winkel-Shirley B. (2001). Flavonoid biosynthesis. A colorful model for genetics, biochemistry, cell biology, and biotechnology. Plant Physiol. 126, 485–493. 10.1104/pp.126.2.485 11402179 PMC1540115

[B37] Yang G. Liang K. Zhou Z. Wang X. Huang G. (2020). UPLC-ESI-MS/MS-based widely targeted metabolomics analysis of wood metabolites in teak (*Tectona grandis*). Molecules 25:2189. 10.3390/molecules.25092189 32392900 PMC7249157

[B38] Yu X. Chu M. Chu C. Du Y. Shi J. Liu X.. (2020). Wild rice (*Zizania* spp.): a review of its nutritional constituents, phytochemicals, antioxidant activities, and health-promoting effects. Food Chem. 331:127293. 10.1016/j.foodchem.2020.127293 32554311

[B39] Yu G Fang Y. Liu J. (2018). Practice and exploration of mulberry leaf edible development and utilization (in Chinese). Bull. Sericult. 49, 49–50. 10.3969/j.issn.0258-4069.2018.03.016

[B40] Zhang L. (2015). Dynamic analysis of constituents in plants of Genus Morns L. in China and study on the suitable drying processing of mulberry leaves. [master's thesis]. Nanjing University of Chinese Medicine. (in Chinese), China.

[B41] Zhang R. Zhang B. He T. Yi T. Yang J. He B. (2016). Increase of rutin antioxidant activity by generating Maillard reaction products with lysine. Bioorganic Med. Chem. Lett. 26, 2680–2684. 10.1016/j.bmcl.2016.04.008 27106712

[B42] Zhao H. Wang J. Lv H. Zhang L. (2020). Effects of drying methods on the main active ingredients and the antioxidant activity of mulberry leaves. Food Industry. 41, 191–195. Available online at: https://kns.cnki.net/kcms/detail/detail.aspx?FileName=SPGY202008048

[B43] Zhao J. Davis L. C. Verpoorte R. (2005). Elicitor signal transduction leading to production of plant secondary metabolites. Biotechn. Adva. 23, 283–333. 10.1016/j.biotechadv.2005.01.003 15848039

