# Supplementary material for: Flavonoid Levels and Antioxidant Capacity of Mulberry Leaves: Effects of Growth Period and Drying Methods
Source: Front Plant Sci. 2021 Aug 4;12:684974. doi: 10.3389/fpls.2021.684974 (PMC8371438; doi:10.3389/fpls.2021.684974)
Supplement: Supplementary file 1 [file Data_Sheet_1.pdf]

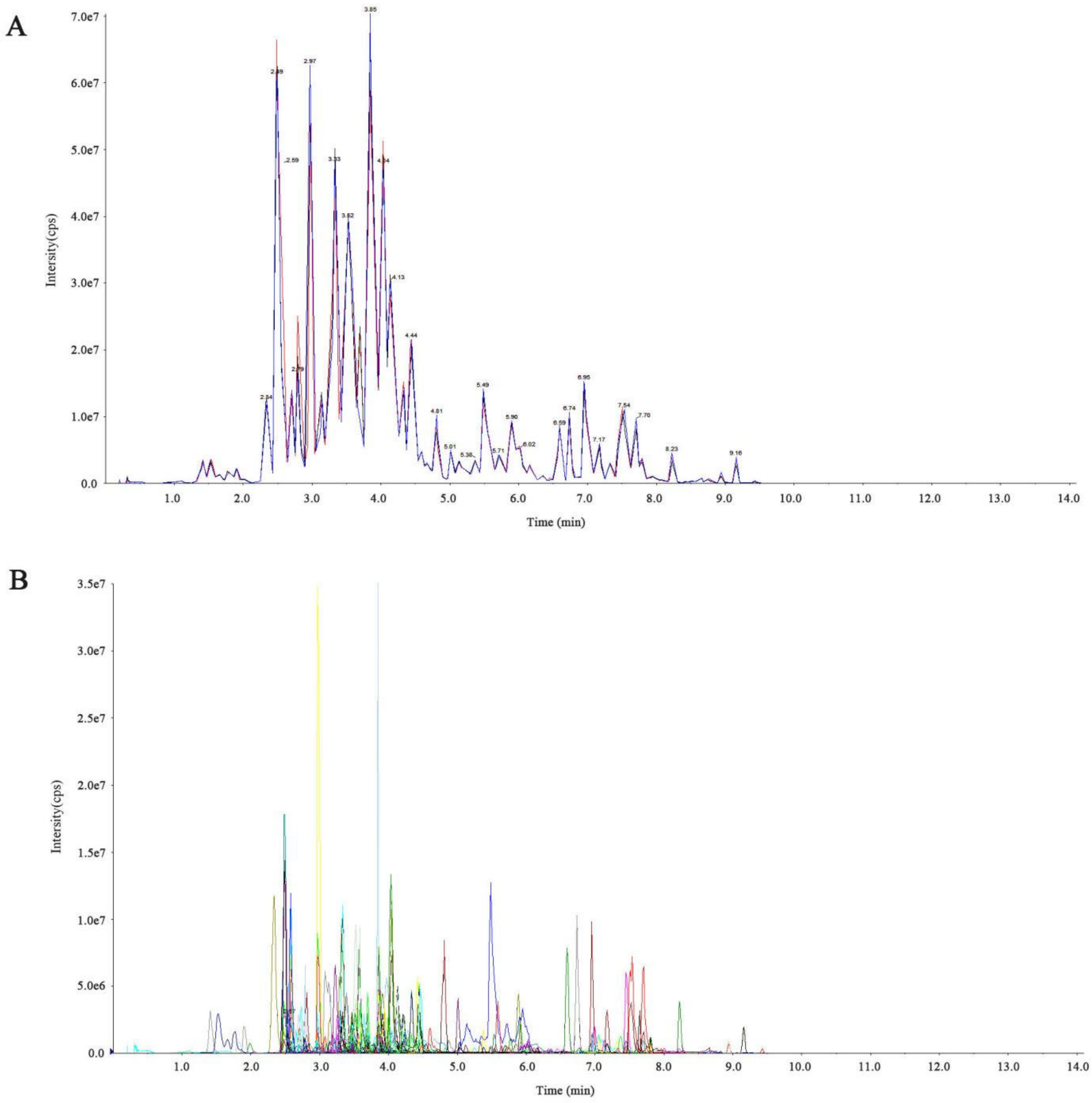

Fig. S1 Total ion current of one quality control sample by mass spectrometry detection **(A)** and multi-peak detection plot of metabolites in the multiple reaction monitoring mode **(B)**.

Table S1. Dry matter content and equilibrium moisture content of mulberry leaves dried by four different methods in YL and OL

Drying method	Dry matter content (%)	Equilibrium moisture content (%)
YL-OD	$22.82 \pm 0.75^{f*}$	4.27 ± 0.27^{cd}
YL-FD	23.91 ± 0.38^{e}	3.86 ± 0.35^{d}
YL-AD	25.50 ± 0.68^{d}	6.04 ± 0.16^{a}
YL-SD	24.40 ± 0.15^{e}	5.10 ± 0.14^{b}
OL-OD	36.59 ± 0.25^{c}	3.07 ± 0.24^{e}
OL-FD	37.46 ± 0.85^{bc}	2.23 ± 0.35^{f}
OL-AD	39.06 ± 0.53^{a}	5.46 ± 0.25^{b}
OL-SD	38.01 ± 0.26^{b}	4.53 ± 0.30^{c}

n = 3

*: Different letters within the same columns denote significant differences between groups ($P < 0.05$).
